# Hnf4α Is Involved in LC-PUFA Biosynthesis by Up-Regulating Gene Transcription of Elongase in Marine Teleost *Siganus canaliculatus*

**DOI:** 10.3390/ijms19103193

**Published:** 2018-10-16

**Authors:** Yuanyou Li, Xiaowei Zeng, Yewei Dong, Cuiying Chen, Cuihong You, Guoxia Tang, Junliang Chen, Shuqi Wang

**Affiliations:** 1 School of Marine Sciences, South China Agricultural University, Guangzhou 510642, China; sunshinewonder@163.com; 2 Guangdong Provincial Key Laboratory of Marine Biotechnology, Shantou University, Shantou 515063, China; zengxiaowei2018@163.com (X.Z.); ccy115@163.com (C.C.); chyou@stu.edu.cn (C.Y.); tangguoxia19870914@163.com (G.T.); 11707006@zju.edu.cn (J.C.); 3 STU-UMT Joint Shellfish Research Laboratory, Shantou University, Shantou 515063, China

**Keywords:** transcription regulation, LC-PUFA biosynthesis, promoter, Hnf4α, Elovl5, Rabbitfish *Siganus canaliculatus*

## Abstract

The rabbitfish *Siganus canaliculatus* is the first marine teleost shown to be able to biosynthesize long-chain polyunsaturated fatty acids (LC-PUFA) from C18 PUFA precursors catalyzed by two fatty acyl desaturases (fad) including Δ4 Fad and Δ6/Δ5 Fad as well as two elongases (Elovl4 and Elovl5). Previously, hepatocyte nuclear factor 4α (Hnf4α) was demonstrated to be predominant in the transcriptional regulation of two *fads*. To clarify the regulatory mechanisms involved in rabbitfish lipogenesis, the present study focused on the regulatory role of Hnf4α to *elovl5* expression and LC-PUFA biosynthesis. Bioinformatics analysis predicted two potential Hnf4α elements in *elovl5* promoter, one binding site was confirmed to interact with Hnf4α by gel shift assays. Moreover, overexpression of *hnf4α* caused a remarkable increase both in elovl5 promoter activity and mRNA contents, while knock-down of hnf4α in *S. canaliculatus* hepatocyte line (SCHL) resulted in a significant decrease of *elovl5* gene expression. Meanwhile, *hnf4α* overexpression enhanced LC-PUFA biosynthesis in SCHL cell, and intraperitoneal injection to rabbitfish juveniles with Hnf4α agonists (Alverine and Benfluorex) increased the expression of *hnf4α*, *elvol5* and Δ4 *fad*, coupled with an increased proportion of total LC-PUFA in liver. The results demonstrated that Hnf4α is involved in LC-PUFA biosynthesis by up-regulating the transcription of the *elovl5* gene in rabbitfish, which is the first report of Hnf4α as a transcription factor of the *elovl5* gene in vertebrates.

## 1. Introduction

Long-chain polyunsaturated fatty acids (LC-PUFA) such as eicosapentaenoic acid (EPA; 20:5n-3), arachidonic acid (ARA; 20:4n-6) and docosahexaenoic acid (DHA; 22:6n-3) are cell membranes components, precursors of lipogenesis [1]. As signal molecules involved in metabolic pathways, LC-PUFAs are also very important to human health, which could respond to immune and inflammatory stimulation [2,3,4]. Fish are the major sources of n-3 LC-PUFAs from the human diet [5], while marine teleost mainly relies on feed rich in fish oil (FO) or fish meal (FM) to meet their requirement for LC-PUFA such as ARA, EPA and DHA. With an increase of global fish consumption, the finite ocean fishery resources led to a rise in the prices of FO and FM, which has greatly impacted the healthy and sustainable development of fish culture [6]. Up to the present day, freshwater fish and salmonid species can convert C18 PUFAs into LC-PUFAs through a series of desaturation and elongation steps catalyzed by fatty acid desaturases (Fad) and elongases of very long-chain fatty acids (Elovl), while most marine teleost are inefficient in LC-PUFA biosynthesis in vivo or lack such a capability [7]. Therefore, the better option for us to relieve such a contradiction is searching for alternatives to fish oil, for instance, terrestrial vegetable oil (VO), which is rich in C18 PUFAs but less rich in LC-PUFAs. However, the VO replacement reduced the LC-PUFA content in muscle and triggered a sub-healthy state in some marine fish [8], and scientists have started to deal with the FO replacement issue from the regulatory mechanism of the key enzyme involved in LC-PUFA biosynthesis. 

As for the teleost *elovl5* gene, it has been cloned and functionally characterized in about twenty fish species [9], and its regulatory mechanism in marine teleost lipogenesis has been reported mainly at the transcriptional level [10]. While in mammals, the regulatory mechanism occurs both at the transcriptional and post transcriptional level. It has been shown that VO treatment up-regulated the expression of Δ6 and Δ5 *fad* as well as some elongases by about 2 to 3-fold when compared with FO feed group, which subsequently led to an increase of LC-PUFA biosynthesis in the liver and intestinal tissues of salmonids [11,12,13,14,15,16]. The up-regulation of *fad* and *elovl* in fish by VO might be due to relief on suppressed gene expression exerted by dietary LC-PUFA, especially DHA [17,18]. SREBP-1 is the main transcription factor involved in such a feedback regulatory process [19,20], while LXR is described as another regulator of *elovl5* in fish [21,22,23]. Recently, Laying Hens and his group demonstrated that estrogen could promote hepatic LC-PUFA biosynthesis by regulating Elovl5 at post-transcriptional level, suggesting that there were different regulatory mechanisms between mammals and teleost [24].

Rabbitfish *S. canaliculatus* is an economically important aquaculture species and the first marine teleost demonstrated by our group to have the LC-PUFA biosynthetic ability from C18 precursors [25]. In addition, all the key enzymes for LC-PUFA biosynthesis including the Δ4 fatty acyl desaturase (Fad) (the first report in vertebrates), Δ6/Δ5 bifunctional Fad (the first report in marine fish) and two elongases of very long-chain fatty acids (Elovl4 and Elovl5) were characterized in this species, thus rabbitfish is a good model for us to study the regulatory mechanisms involved in LC-PUFA biosynthesis of teleosts [26,27]. Recently, we have characterized Δ4 Fad promoter structure and identified that hepatocyte nuclear factor 4α (HNF4α) was involved in the transcription regulation of Δ4 Fad gene, which was the first demonstration of HNF4α as a transcription factor (TF) of vertebrate Fad gene [28,29,30]. To clarify the overall regulatory mechanisms of LC-PUFA biosynthesis in rabbitfish, the present study focused on the promoter analysis of *elovl5* gene and regulatory role of HNF4α to that gene. The promoter sequence of *elovl5* was cloned by genome walking, and bioinformatic software was used to predict a possible HNF4α element, moreover, the regulatory role of such TF in rabbitfish LC-PUFA biosynthesis was confirmed by overexpression (pcDNA3.1+HNF4α and mRNA), RNAi and agonist assay. The results could increase our understanding of the regulatory mechanisms of LC-PUFA biosynthesis in vertebrates, which would also contribute to the optimization and/or enhancement of the LC-PUFA pathway in teleosts.

## 2. Results

### 2.1. The Basic Structure of Rabbitfish Elovl5 Gene Promoter

**Figure 1:**
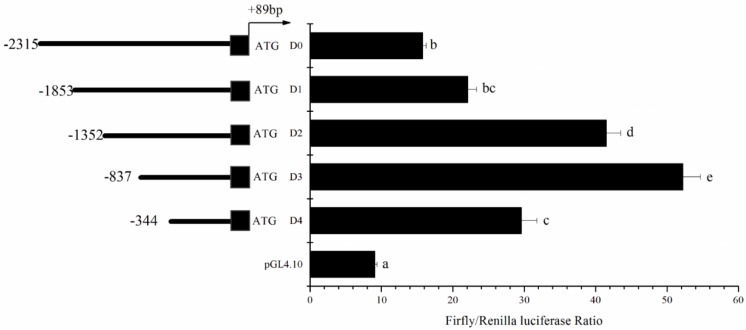
Structure analysis of the 5′ flanking sequence of *S. canaliculatus elovl5* by deletion. Deletion constructs are represented on the left. Non-coding exons are shown by black boxes. The intron is indicated with a black line between the two exons. The sequence is numbered relative to the transcription start site (TSS), which is defined as the first base of the first 5′ non-coding exon. Promoter activity of each construct is represented with normalized value (Firefly luciferase: Renilla luciferase) on the right. Results are means ± SEM (*n* = 3). Values in each row not sharing a common letter indicate significant difference (analyzed by ANOVA followed by paired *t*-test; *p* < 0.05).

**Figure 2:**
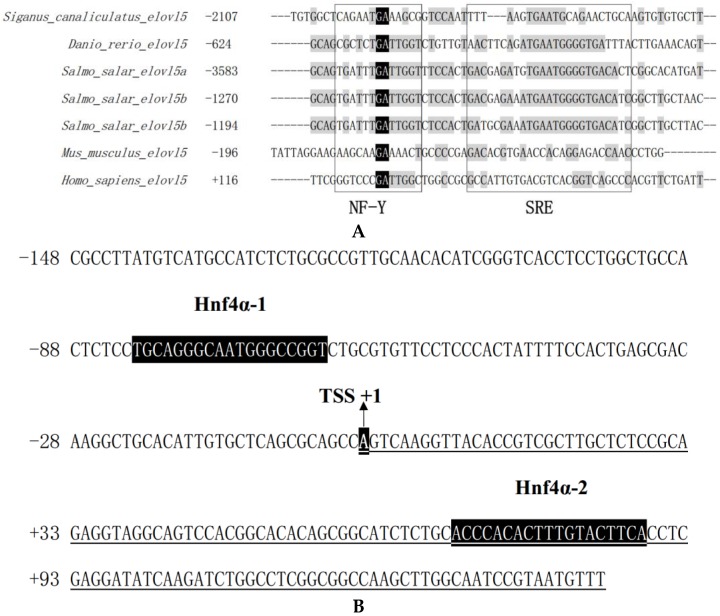
The promoter structure of rabbitfish *elovl5*. (**A**) Alignment for the conserved elements of NF-Y and SRE in *elovl5* promoter region. (**B**) The position of Hnf4α element in rabbitfish *elovl5* promoter region, which is relative to the transcription start site (TSS, +1). The bases with black background refer to Hnf4α-1, TSS, and Hnf4α-2, respectively. The bases underlined are downstream sequence of TSS.

The upstream sequence of 3323 bp from the initiation codon ATG of *elovl5* was cloned and the region between −2315 bp ~ +89 bp was determined as the possible promoter region, while the first base of the first non-coding exon was regarded as the putative TSS, its position was defined as the +1 in the sequence. Based on equal progressive deletion of the 5′ flanking sequence of *elovl5*, the mutants including D0 (−2315 bp to +89 bp), D1 (−1852 bp to +89 bp), D2 (−1351 bp to +89 bp) and D3 (−837 bp to +89 bp) were shown to cause a gradual increase in promoter activity. However, a significantly reduced promoter activity occurred with D4 (−344 bp to +89 bp), while the highest promoter activity occurred with D3 (−837 bp to +89 bp), suggesting that the core promoter region was from −837 bp to −344 bp (Figure 1). And very low promoter activity was detected in the negative control D0 (pGL4.10) (Figure 1). The conserved elements of NF-Y and SRE were identified by alignment with several other promoter sequences; that unit was about −2107 bp to TSS (Figure 2A).

### 2.2. Two Hnf4α Binding Sites Were Predicted in Rabbitfish Elovl5 Promoter

Using the bioinformatics software TRANSFAC^®^ and TF binding^®^, two Hnf4α binding sites (+70 ~ +81 and −84 ~ −74) were predicted in the promoter region of D3 (−837 bp to +89 bp) of rabbitfish *elovl5* (Table 1 and Figure 2B). Based on these results, we speculated that Hnf4α might be a potential factor that affects the activity of the rabbitfish *elovl5* promoter.

### 2.3. Overexpression of Hnf4α Increased Elvol5 Promoter Activity

**Figure 3:**
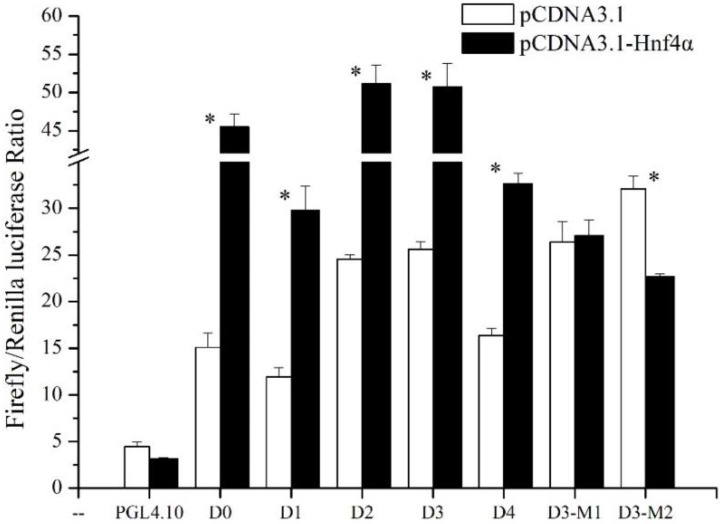
Effects of *S. canaliculatus hnf4α* over-expression on activity of *elovl5* promoter deletion mutants and site-directed mutation in HEK 293T cells. The *elovl5* promoter deletion mutants, site-directed mutants and negative control were co-transfected with the overexpression plasmid pcDNA3.1-Hnf4α, while the control group was transfected with the empty vector pcDNA3.1. The negative control pGL4.10 is an empty vector with no promoter sequence upstream the reporter gene. Each plasmid complex was transfected in triplicate in three independent experiments. Significant differences compared with the corresponding control group were analyzed using Student’s *t*-test; with * denoting *p* < 0.05.

To explore the regulatory role of rabbitfish Hnf4α in *elvol5* gene transcription, the effect of rabbitfish *hnf4α* overexpression on *elvol5* promoter activity was determined. The recombinant plasmid pcDNA3.1-Hnf4α and progressive deletion mutants of *elvol5* promoter or site-directed mutants were co-transfected into HEK 293T cells. The promoter activity of each progressive deletion mutant significantly increased with *hnf4α* over-expression, while the negative control pGL4.10 showed no response to Hnf4α treatment (*p* < 0.05) (Figure 3). As to the site-directed mutants, D3-M1 showed no response to *hnf4α* overexpression compared with the wild type D3, while the promoter activity of D3-M2 was decreased after *hnf4α* overexpression (Figure 3). 

### 2.4. Electrophoretic Mobility Shift Assay

**Figure 4:**
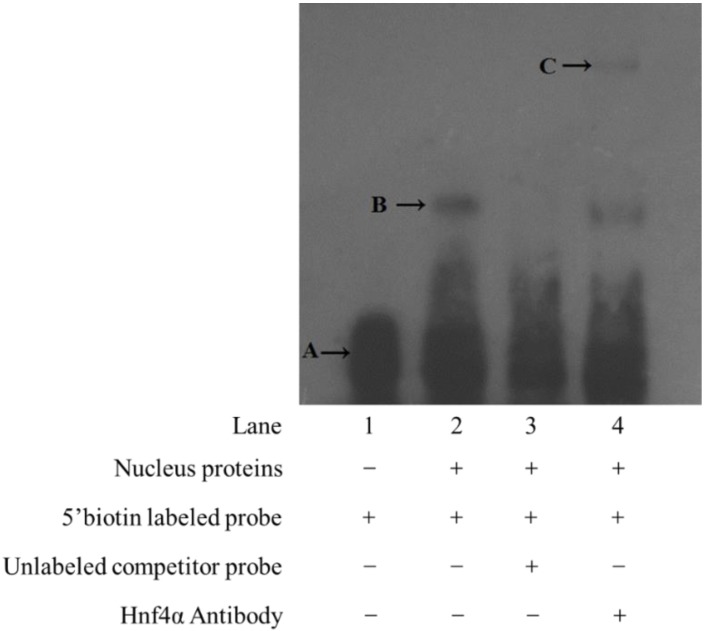
The electrophoretic mobility shift assay (EMSA) of *elovl5* probe with *Siganus canaliculatus* hepatocytes nuclear proteins. Each lane is represented as: Lane 1 (no proteins, 5′ biotin labeled probe), lane 2 (hepatocyte nucleoprotein, 5′ biotin labeled free probe), lane 3 (hepatocyte nucleoprotein, unlabeled competitor probe, 5′ biotin labeled free probe), lane 4 (hepatocyte nucleoprotein, 5′ biotin labeled probe, Hnf4α antibody). Band A is gel shift of DNA-protein complexes. Band B is the free probe. Band C is supershift of DNA-protein-antibody complexes. “+” means that the corresponding material in the row has been added, and “−” means that the material is not added.

To further confirm whether Hnf4α in rabbitfish liver could bind to the promoter of *elovl5*, EMSA (electrophoresis mobility shift assay) was performed with rabbitfish hepatocytes cytoplasmic and nuclear proteins. The results indicated that the hepatocytes nuclear proteins bound to biotin-labeled probe and retarded their mobility (Figure 4 lane 2). When the assays were further performed using unlabeled probe (Figure 4 lane 3) as a specific competitor, the specific shift was abolished by excess unlabeled probe, which indicated specific binding of hepatocytes nuclear proteins to the probe (Figure 4). The specific binding of Hnf4α to *elovl5* was also confirmed by its super shift after the addition of Hnf4α antibody (Figure 4 lane 4). These results further suggested that Hnf4α specifically bound to the predicted binding sites (D3-M1) in the upstream of *elovl5* and thereby might regulate *elovl5* transcription. 

### 2.5. Overexpression of Hnf4α Enhanced Elvol5 Gene Expression and LC-PUFA Biosynthesis in SCHL Cells

**Figure 5:**
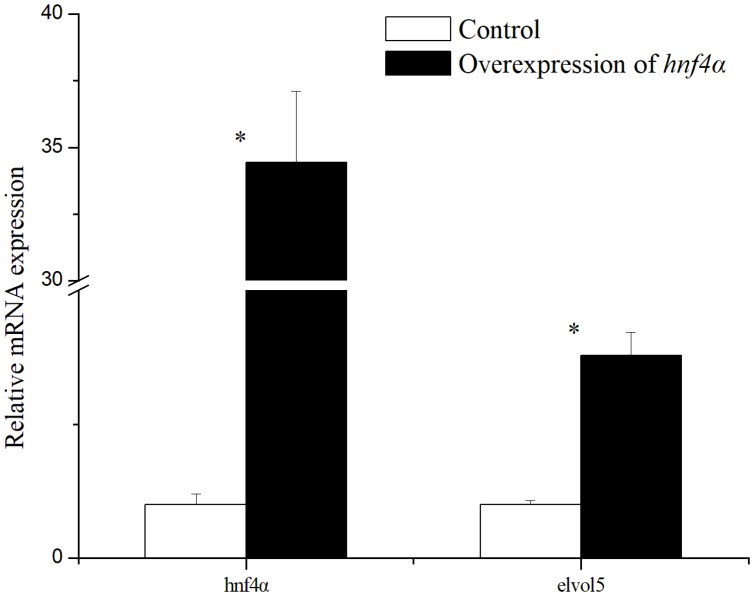
Q-PCR analysis of *hnf4α* and *elovl5* gene expression level in SCHL cells transfected with *hnf4α* mRNA or control. The Relative expression of *hnf4α* and *elovl5* were analyzed by qPCR and normalized to 18S rRNA expression using the by 2^−ΔΔ*C*t^ method. Results are means ± SEM (*n* = 3). Significant difference compared with the control group was analyzed using Student’s *t*-test; with * denoting *p* < 0.05.

**Figure 6:**
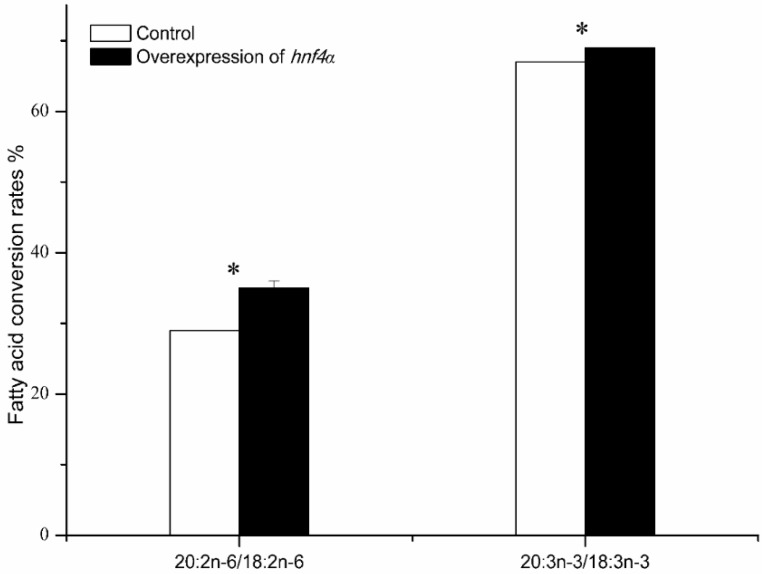
Fatty acid conversion rates in SCHL cells transfected with *hnf4α* mRNA compared with control. White columns represent the control groups while the black columns are the experiment groups transfected with *hnf4α* mRNA. Results are means ± SEM (*n* = 3). Significant differences compared with the control group were analyzed using Student’s *t*-test; with * denoting *p* < 0.05.

To further confirm the regulatory role of rabbitfish Hnf4α in *elvol5* gene transcription, rabbitfish *hnf4α* mRNA synthesized in vitro was transfected into SCHL cells. The mRNA expression level of *hnf4α* and *elvol5* was determined by qPCR, with the results showing that the mRNA levels of *hnf4α* and *elovl5* significantly increased after *hnf4α* mRNA transfection (Figure 5). We therefore analyzed the effect of *hnf4α* overexpression on fatty acids composition in the SCHL cells. The results from this analysis showed that the levels of ARA, EPA and DHA were significantly up-regulated (Table 2), and the conversion rates of 18:2n-6 to 20:2n-6 and 18:3n-3 to 20:3n-3, the two pathways catalyzed by Elovl5, were enhanced after *hnf4α* overexpressing (Figure 6).

### 2.6. Knockdown of Hnf4α Expression Reduced Elvol5 Expression in SCHL Cells

**Figure 7:**
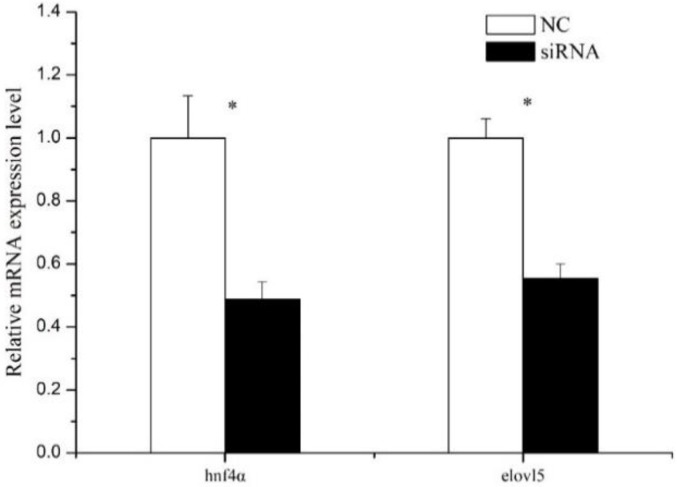
Q-PCR analysis of *hnf4α* and *elovl5* gene expression level in rabbitfish hepatocytes transfected with *hnf4α* siRNA or control siRNA (NC). The relative expression of *hnf4α* and *elovl5* were analyzed by qPCR and normalized to 18S rRNA expression using the by 2^−ΔΔ*C*t^ method. Results are means ± SEM (*n* = 3). Significant differences compared with the control group were analyzed using Student’s *t*-test; with * denoting *p* < 0.05.

RNA interference assay was carried out so as to further investigate the regulatory role of Hnf4α on *elvol5* gene expression in SHCL cells. First, the efficiency of the siRNA to silence *hnf4α* was evaluated by analyzing the mRNA levels of *hnf4α* using qPCR. The results indicated that the mRNA level of *hnf4α* was significantly down-regulated by about 51.1% at 24 h after *hnf4α* siRNA transfection (Figure 7). Meanwhile, the mRNA expression level of *elovl5* decreased by about 44.5% compared with negative control group (NC) (Figure 7). 

### 2.7. Intraperitoneal Injection of Hnf4α Agonists Increased Elvol5 and Δ4 Fad Expression and Fatty Acid Composition in Rabbitfish Liver

**Figure 8:**
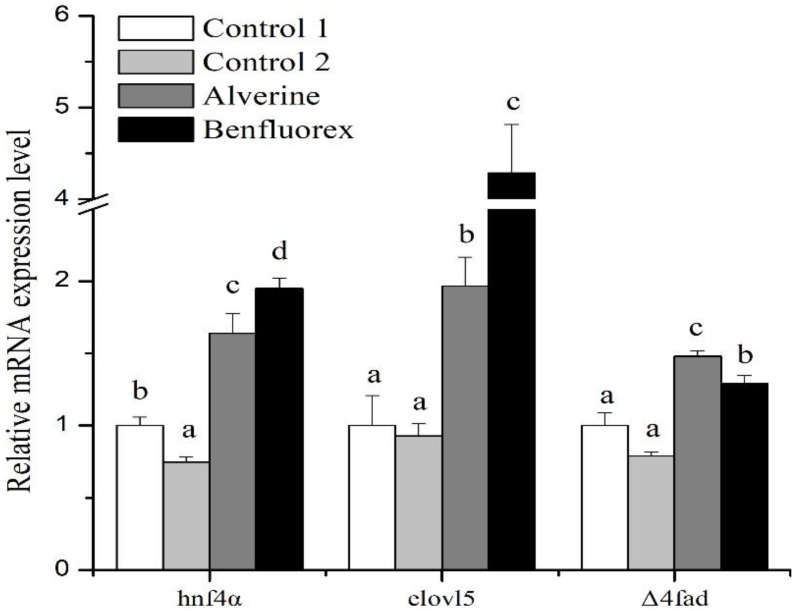
Q-PCR analysis of *hnf4α*, *elovl5* and ∆4 *fad* gene expression level in liver of juvenile rabbitfish injected with Hnf4α agonists (Alverine and Benfluorex) or control. The relative expression of *hnf4α*, *elovl5* and ∆4 *fad* was analyzed by qPCR and normalized to 18S rRNA expression using the by 2^−ΔΔCt^ method. Control 1 was injected with 0.9% NaCl while control 2 was injected with 2.5% DMSO. Results are means ± SEM (*n* = 6). Significant differences were analyzed by ANOVA followed by Tukey’s multiple comparison test; with * denoting *p* < 0.05.

To further identify the regulatory role of *hnf4α* on rabbitfish LC-PUFA biosynthesis *in vivo*, Hnf4α agonists (Alverine and Benfluorex) were injected into the enterocoelia of juvenile rabbitfish. Real time qPCR results in liver samples showed that the gene expression levels of *hnf4α*, *elovl5* and Δ4 *fad* significantly increased in Alverine and Benfluorex treatment groups compared to the negative control (Figure 8). Moreover, the results of fatty acid composition in liver showed that there was a higher content of DHA and total HUFA in Alverine injection group compared with the negative control (Table 3), and the content of EPA in Benfluorex treatment group was also higher than that in negative control. 

## 3. Discussions 

To gain insight into the regulatory mechanisms of hepatocyte nuclear factor 4α (Hnf4α) in LC-PUFA biosynthesis of marine teleosts, previous studies we conducted in rabbitfish showed that Hnf4α targeted at Δ4 *fad* and Δ6/Δ5 *fad* promoter directly and upregulated their gene expression [28,29,30]. Above all, such TF has been considered as one vital regulator involved in LC-PUFA biosynthesis. However, the influence of Hnf4α to of *elovls* gene transcription has not been studied and whether Hnf4α could directly regulate *elovl5* expression was still unknown. Therefore, the present study focused on the regulatory role of Hnf4α in *elovl5* gene transcription and LC-PUFA biosynthesis of rabbitfish *S. canaliculatus*.

Hnf4α is an important regulator of the key enzymatic genes involved in vertebrates LC-PUFA biosynthesis. As a vital TF involved in the regulation of lipid and cholesterol metabolism, HNF4α has been reported to activate the following targets: Apolipoprotein C-III (ApoCIII), Cholesterol 7α (Cyp7α) Hydroxylase [31,32], fatty acid synthase (FAS) [33], stearoyl-CoA desaturase (SCD) and Δ4 Fad [28]. Recently, one study identified a fragment of HNF4α binding to the core promoter of rabbitfish Δ6/Δ5 *fad*, suggesting its potential modulation to this new target [29], while another study demonstrated that Hnf4α is involved in the transcriptional regulation of LC-PUFA biosynthesis by targeting Δ4 *fad* and Δ6/Δ5 *fads* in rabbitfish [30]. *elovl5* is another key enzymatic gene in LC-PUFA biosynthesis, which has attracted many researchers with lots of studies focused on its transcriptional mechanism in humans, mice and salmon. At present, SREBPs have been demonstrated as the major regulator in *elovl5* transcription, and LXR might be another potential TF involved in such a process directly in salmon, while in mammals it was an indirect TF that influenced *elovl5* expression [22,34]. The present study discovered a conservative element unit of NF-Y and SRE in *elovl5* promoter, which was similar to the previous reports mentioned above, suggesting that SREBPs might be the main regulator in rabbitfish *elovl5* transcription. Additionally, we have also identified the positive effect of Hnf4α on rabbitfish *elovl5* expression through site-directed mutation, electrophoretic mobility shift assay, *hnf4α* overexpression, *hnf4α* knock-down by RNAi, as well as by drug treatment. The results were remarkable as this novel discovery expanded the regulatory range of Hnf4α target genes in lipogenesis. Thus, with the addition of *elovl5*, Hnf4α has now been demonstrated to positively regulate the complete enzymatic pathway in LC-PUFA biosynthesis (FAS, Fad, Elovl), suggesting its prominent role in lipid metabolism. 

HNF4α could improve LC-PUFA biosynthesis in SCHL cells by increasing *elovl5* gene expression. In general, HNF4α acted as a positive regulator in lipogenesis, as its feed-back regulation in energy metabolism maintains physiological homeostasis in organisms [35]. As a ligand-dependent TF, long chain fatty acids such as ALA, EPA and DHA are endogenous ligands for HNF4α, so binding to this nuclear receptor suppressed its activation to the target genes [36]. Some chemical ligands such as those used in the present study, i.e., Alverine and Benfluorex, could increase *hnf4α* gene expression and activate this TF as a ligand [37]. In relation to recent research on the regulation of *elovl5* in the teleost *Larimichthys crocea* and *Epinephelus coioides*, feed-back regulation in LC-PUFA biosynthesis from the process of dietary lipid to fish metabolism and LC-PUFA (EPA and DHA) to hepatocytes was carried out through another nuclear receptor LXRα and its downstream target SREBP-1 [21,23]. Previous research in Salmon and SHK-1 cell lines also supported this regulation pattern at both the nutritional and cellular level [22,34]. The present study tested the feedback regulation model of Hnf4α in rabbitfish from the physiological level with chemical ligands and at the cellular level with fatty acid substrate conversion. The results indicated that Alverine and Benfluorex could activate *hnf4α* and *elovl5* gene expression and then improve LC-PUFA biosynthesis in rabbitfish, while *hnf4α* mRNA overexpression revealed that *hnf4α* overexpression could improve LC-PUFA biosynthesis in SCHL cells. This observation further demonstrates the important role of *hnf4α* in rabbitfish LC-PUFA biosynthesis, which is a completely novel mechanism in vertebrate lipogenesis.

In conclusion, the *elovl5* promoter of *S. canaliculatus* was cloned and characterized, moreover Hnf4α was demonstrated to be a TF of *elovl5* in vertebrate for the first time, both discoveries have suggested a new regulatory mechanism of LC-PUFA biosynthesis in teleost.

## 4. Materials & Methods

### 4.1. Compliance with Ethical Standards

In the present study, we followed the requirement of the National Institutes of Health guide (NIH Publications No. 8023, revised 1978) and the Institutional Animal Care and Use Committee of Shantou University (Academic Behavior Criterion of Shantou University, [2014]-6, 17 January 2014) to treat rabbitfish with 0.01% 2-phenoxyethanol (Sigma-Aldrich, St. Louis, MO, USA) anesthesia. The rabbitfish were obtained from wild environments near the coast of the NanAo Marine Biology Station.

### 4.2. Cloning of 5′ Flanking Sequence of Rabbitfish Elovl5

Genomic DNA was extracted from rabbitfish muscle with the proteinase K and phenol method as previously noted [38]. The Genome Walker^TM^ Universal Kit (TaKaRa Bio, Tokyo, Japan) was used for *elovl5* promotor cloning according to the manufacturer’s instructions. Nested PCR was performed with the outer adaptor primer AP1 in the kit and a specific antisense primer E5UA0, while the secondary nested PCR reaction was carried out with the nested adaptor primer AP2 and specific antisense primer E5UA1 (Table 4). The primers E5UA0 and E5UA1 were designed based on the mRNA sequence of *elovl5* (GenBank: GU597350.1) [27]. After two rounds of PCR, the PCR product of the upstream sequence was recovered and isolated by gel extraction, then inserted into the pMD18-T Vector (TaKaRa Bio, Tokyo, Japan), and sequenced (Sangon Biotech Co., Ltd., Shanghai, China). The sequencing results revealed the presence of first non-coding exons in the 5′ untranslated region (UTR) of *elovl5*, indicating that the PCR product was indeed the 5′ flanking sequence of *elovl5*.

### 4.3. Bioinformatics Analysis

The conserved elements of NF-Y and SRE in rabbitfish *S. canaliculatus elovl5* promoter were identified by alignment with the corresponding *elovl5* promoter sequence from *Salmo_salar* (GU238431.1 and GU324549.1), *Danio_rerio* (NC_007124.7), *Mus_musculus* (NC_000075.6), and *Homo_sapiens* (NG_034263.1). Online software including JASPAR^®^, TRANSFAC^®^ and TF Binding^®^ were used to analyze the promoter region of *elovl5* for potential TF binding sites. The potential TF elements were obtained from the predicted results analyzed by the software.

### 4.4. Identification of Elovl5 Core Promoter through Progressive Deletion Mutation

To identify the core promoter region within the cloned 5′ flanking sequence of rabbitfish *elovl5*, the candidate promoter was progressively deleted. PCR reaction was carried out using 2× *pfu* PCR Master Mix (Tiangen Biotech, Beijing, China) with genomic DNA as template with forward primers (E5D0, E5D1, E5D2, E5D3, E5D4) containing a 5′ *Kpn*I site and the antisense primer SigE5UA1 containing a *Xho*I site to obtain the full-length promoter fragment (D0: 2404 bp) and four deletion mutant fragments (D1, 1942 bp; D2, 1441 bp; D3, 926 bp; D4, 433 bp) (Figure 1). PCR products were digested by the restriction endonucleases *Kpn*I and *Xho*I (New England Bio labs, Ipswich, UK) and inserted into the pGL4.10 [luc2] vector (Promega, Madison, WI, USA). The upstream sequence in the insert fragments D0, D1, D2, D3 and D4 had lengths −2315 bp, −1853 bp, −1352 bp, −837 bp and −344 bp, respectively relative to the putative transcription start site (TSS) +1. The TSS was predicted as the first base of the first non-coding exon (Figure 1). After construction, the vector consisted of insert fragments (D0, D1, D2, D3 and D4) and pGL4.10, and high Pure Plasmid Isolation Kit (Roche, Mannheim, Germany) was used to isolate the construct. Later, the transfection assay in human embryonic kidney (HEK293T) cells (Chinese Type Culture Collection, Shanghai, China) was carried out.

### 4.5. Functional Identification of the Two-Candidate Hnf4α Elements

To determine the potential effect of the predicted Hnf4α binding sites on promoter activity, recombinant plasmids with site-directed mutation of Hnf4α elements in the promoter was constructed. For the rabbitfish *elovl5* promoter, deletion mutant D3 containing core promoter region was treated as wild-type and site-directed mutants were produced from this using the Muta-direct^TM^ site-directed mutagenesis kit (SBS Genetech, Shanghai, China) according to the manufacturer’s instruction. The strategy of site directed mutation is shown in Table 1 and the primers are shown in Table 4. The site-directed mutation plasmids from D3 are designated D3-M1 and D3-M2. The over-expression plasmid pcDNA3.1-Hnf4α contains the whole Open reading frame (ORF) of rabbitfish Hnf4α. All the recombinant plasmids were isolated with High Pure Plasmid Isolation Kit (Roche, Swiss) for use in transfection. HEK 293T cells were seeded onto 96-well plates at a density of 4 × 10^4^ per well in a volume of 100 μL per well with High Glucose Dulbecco’s Modified Eagle Medium (DMEM) (Gluta MAX) (Gibco, Thermo Fisher, Carlsbad, CA, USA) and 10% fetal bovine serum (Gibco, Life Technologies, Carlsbad, CA, USA), then cultured at 37 °C. Transfection was carried out with mutants of *elvol5* promoter including D0, D1, D2, D3, D4, D3-M1, D3-M2 (100 ng/well), pGL4.75 (0.02 ng/well) and pcDNA3.1-Hnf4α (50 ng/well), with pGL4.10 used as vector control, following the method described previously [28]. Transfections were done in triplicates and three independent experiments. Cell culture medium was replaced with 75 μL DMEM + 10% FBS at 24 h after transfection. Luciferase assays were performed at 48 h after transfection with the Dual-Glo^TM^ luciferase assay system (Promega, Madison, WI, USA), and luminescence was detected by a microplate reader (InfiniteM200 Pro, Tecan, Switzerland). The method for promoter activity calculation was the same as previously noted [28].

### 4.6. Electrophoretic Mobility Shift Assay (EMSA)

To confirm the binding of Hnf4α to the promoter of rabbitfish *elovl5*, nuclear and cytoplasmic proteins were extracted from rabbitfish hepatocytes with the Beyotime Nuclear Extract Kit (Beyotime Institute of Biotechnology, Haimen, China) and quantified by Modified BCA Protein Assay Kit (Sangon, Shanghai, China). The 29 bp 5′ end biotin-labeled probe covering the predicted Hnf4α elements was designed and incubated with the proteins to determine whether Hnf4α interacted with the promoter of *elovl5*. Both the labeled and unlabeled probes in the experiment were obtained from Shanghai Sangon Biotech Co., Ltd., while the EMSA reaction system was performed with the Beyotime Chemiluminescent EMSA Kit (Beyotime Institute of Biotechnology, Haimen, China) according to the manufacturer’s instructions. For the super shift assay, 1 μL antibody (Abcam, Cambridge, MA, USA) of Hnf4α was pre-incubated with nuclear or cytoplasmic proteins for 30 min at 0–4 °C. Samples obtained after the binding reaction were subjected to a 4% non-denaturing polyacrylamide gel electrophoresis and transferred onto a nylon membrane. The 5′ end biotin-labeled probe was detected using a streptavidin-horseradish peroxidase conjugate and a chemiluminescent substrate. The signal was then detected by autoradiography with X-OMAT BT X-ray film (Kodak, Rochester, MN, USA). 

### 4.7. In Vitro mRNA Transcription of Rabbitfish Hnf4α

The in vitro transcription of *hnf4α* mRNA was performed on a linearized DNA template containing T7 promoter and rabbitfish *hnf4α* cDNA sequence using the mMESSAGE mMACHINE^®^ T7 Ultra Kit (Ambion, Thermo Fisher, Carlsbad, CA, USA). The pcDNA3.1-Hnf4α plasmid previously constructed in our laboratory was used to produce the linearized DNA template [28]. Finally, the product containing the *hnf4α* mRNA was purified with MEGAclear^TM^ Kit (Ambion, Austin, TX, USA) and used immediately or stored at −80 °C for later use.

### 4.8. Transfection of Rabbitfish Hnf4α mRNA and siRNA into SCHL Cells

The rabbitfish *S. canaliculatus* hepatocytes cell line (SCHL) established by our group [39] were seeded onto 6-well plates (Eppendorf, Hamburg, Germany) at a density of 1.2 × 10^6^ per well in a volume of 2 mL Dulbecco’s modified Eagle’s medium (DMEM)-F12 medium (Gibco, Life Technologies, Carlsbad, CA, USA) supplemented with 10% foetal bovine serum (FBS) (Gibco, Life Technologies, Carlsbad, CA, USA) and 0.5% rainbow trout *Oncorhychus mykiss* serum (Caisson Labs; www.caissonlabs.com), and maintained at 28 °C. At 80% confluence, cells were transfected with 5 μg/well *hnf4α* mRNA using Lipofectamine^TM^ Messenger-MAX^TM^ Reagent (Thermo Fisher, Carlsbad, CA, USA). Medium was removed at 48 h post transfection, cells washed carefully with 1 mL PBS, and then total RNA extracted using 1 mL Trizol (Invitrogen, Carlsbad, CA, USA). For lipid extraction and fatty acids content detection, the SCHL cells were seeded onto six 100 mm dishes (Eppendorf, Hamburg, Germany) at a density of 7 × 10^6^ cells per well in a volume of 8 mL (DMEM/F12 + 10% FBS + 0.5% rainbow trout *Oncorhychus mykiss* serum) and maintained at 28 °C. At about 24 h or 70% confluence, cells were then transfected with 6 μg/per dish *hnf4α* mRNA using Lipofectamine^TM^ Messenger-MAX^TM^ Reagent (Invitrogen, Carlsbad, CA, USA). Transfections were done in triplicates and three independent experiments. At 24 h post transfection, medium was replaced with 8 mL DMEM/F12 + 10% FBS + 0.5% rainbow trout *Oncorhychus mykiss* serum, and at 72 h post transfection, cells were treated with 1 mL Trypsin-EDTA (Invitrogen, Carlsbad, CA, USA), centrifuged at 1500× *g* for 2 min, and then fatty acids were extracted from the precipitate as described in Section 4.11. 

In order to knockdown the expression of *hnf4α* in SCHL cells, 21-nucleotide small interfering RNA duplexes (siRNA) targeting *hnf4α* and negative control siRNA (Table 4) were chemically synthesized by Gene-Pharma Biotechnology Company (Suzhou, China). The siRNAs were diluted with DEPC-water to a final concentration of 125 mg/mL. SCHL cells were seeded onto 12-well plates (Eppendorf, Hamburg, Germany) at a density of 5 × 10^5^ cells per well in a volume of 1 mL medium (DMEM/F12 + 10% FBS + 0.5% rainbow trout *Oncorhychus mykiss* serum) maintained at 28 °C, and after 24 h or 60% confluence, cells were then transfected with 40 pmol siRNA per well using Lipofectamine^TM^ 2000 Reagent (Invitrogen, Carlsbad, CA, USA). At 24 h post transfection, media was removed, cells washed carefully with 1 mL PBS, and then total RNA extracted with 1 mL Trizol (Invitrogen, Carlsbad, CA, USA).

### 4.9. Quantitative RT-PCR Assay (Q-PCR)

Total extracted RNA were detected by electrophoresis and quantified by Nanodrop 2000 Spectrophotometer (Thermo Fisher, Carlsbad, CA, USA), followed by cDNA synthesis using High-Capacity cDNA Reverse Transcription Kits (Thermo Fisher Scientific, USA). The expression levels of *hnf4α*, *elvol5*, Δ4 *fad* was determined by Q-PCR using gene specific primers (Table 4) and the relative expression normalized to the reference gene 18S rRNA calculated by the comparative threshold cycle (Ct) method [40]. The Q-PCR reactions were carried out on the Lightcycler 480 system (Roche, Basel, Switzerland). Triplicate wells were used per sample and three independent experiments performed. 

### 4.10. Intraperitoneal Injection Experiments

Eighty healthy juvenile rabbitfish (~30 g each) were captured and randomly divided into four groups (20 per group). They were kept in four indoor seawater tanks (32 ppt) at 25 °C for 4 weeks and fed on FO diets to adapt to the laboratory conditions before further processing. Next, 0.02 g Hnf4α agonist Alverine citrate (Sigma, Ronkonkoma, New York, NY, USA) was dissolved in 10 mL 0.9% NaCl (normal saline, NS) (Sangon Biotech, Shanghai, China) to obtain an operating concentration of 1 mg/mL, while 0.4 g Hnf4α agonist Benfluorex hydrochloride (Sigma, Ronkonkoma, New York, NY, USA) was dissolved in 4 mL dimethyl sulfoxide (DMSO) (Sigma, Ronkonkoma, New York, NY, USA) and diluted 40-fold with 0.9% NaCl as working solution. All fish were fasted at the day before injection. The groups treated with 0.9% NaCl and 2.5% DMSO treatment (1 mL liquid per 100 g fish weight) were set as control respectively, while the groups treated with Alverine citrate (1 mg/mL) and Benfluorex hydrochloride (2.5 mg/mL) were set as experiment groups. For injection, fish were first anaesthetized with 0.01% 2-phenoxyethanol and weighed, and then drugs were slowly injected into their abdominal cavity (1 mL drug per 100 g fish weight). After injection, fish were put back into the aquaculture tanks for recovery. After 24 h, each group was feed, and 48 h post injection, a similar dose of injection was repeated. At 72 h post the first injection, ten fish from each group were anaesthetized with 0.01% 2-phenoxyethanol, liver tissues from each fish collected into tubes, dipped immediately into liquid nitrogen and stored at −80 °C for subsequent extraction of total RNA and lipids.

### 4.11. Lipid Extraction and Analysis by Gas Chromatography-Mass Spectrometer (GC-MS)

For fatty acid extraction, cells or tissue samples were homogenized in chloroform/methanol (2:1, *v*/*v*) with 0.01% 2,6-butylated hydroxytoluene (BHT) as antioxidant, and total lipid was extracted according to the method described by Folch et al. [41]. We used boron trifluoride etherate (ca. 48%, Acros Organics, Thermo Fisher, Carlsbad, CA, USA) to prepare fatty acid methyl esters (FAME) through the reaction of transesterification [25]. FAME were purified by Thin-Layer Chromatography (20 cm × 20 cm × 0.25 mm), resuspended in hexane [42], and separated using a gas chromatograph GC 2010-plus (Shimadzu, Japan) as described by Li et al. [26]. Samples were analyzed in triplicates. GC-MS was used to analyze the fatty acid composition of cell or tissue sample.

### 4.12. Statistical Analysis

All data is presented as means ± SEM. Analysis of data was by one-way analysis of variance (ANOVA) followed by Tukey’s multiple comparison tests or Student’s *t*-test using Origin 7.0 software program. A significance of *p* < 0.05 was applied to all statistical tests performed.

## Figures and Tables

**Table 1:** Hnf4α binding sites predicted using online software and site-directed mutation sites.

TF	Software	Position	Predicted Site	Mutation Site
Hnf4α-1	Comparison	+70 ~ +88	ACCCACACTTTGTACTTCA	ACACTTTGTACT→×
Hnf4α-2	TF binding ®	−84 ~ −64	TGCAGGGCAATGGGCCGGT	GGCAATGGGCC→×

The position of each element is numbered relative to the presumed TSS. The bases underlined are the mutation sites for site-directed mutant, “×” denotes deletion.

**Table 2:** The main fatty acids composition of SCHL cells in over-expressing *hnf4α* group and control group (% area).

Main Fatty Acids	Groups	
	Control	Overexpression of *hnf4α*
14:0	1.37 ± 0.11	1.26 ± 0.01
16:0	14.49 ± 0.21	14.54 ± 0.06
18:0	15.28 ± 0.40	14.89 ± 0.06
24:0	0.71 ± 0.04	0.75 ± 0.01
18:1n-9	21.39 ± 0.56	22.03 ± 0.17
24:1	1.23 ± 0.03	1.16 ± 0.17
18:2n-6	2.69 ± 0.06	2.79 ± 0.03
18:3n-6	0.58 ± 0.06	0.60 ± 0.04
20:2n-6	1.08 ± 0.06 ^a^	1.44 ± 0.05 ^b^
20:4n-6 (ARA)	0.25 ± 0.02	0.35 ± 0.02
18:3n-3	2.41 ± 0.07	2.45 ± 0.04
18:4n-3	0.44 ± 0.02	0.52 ± 0.03
20:3n-3	4.95 ± 0.15 ^a^	5.33 ± 0.03 ^b^
20:5n-3 (EPA)	2.97 ± 0.08 ^a^	3.28 ± 0.01 ^b^
22:5n-3	3.70 ± 0.03 ^a^	3.90 ± 0.01 ^b^
22:6n-3 (DHA)	15.39 ± 0.43 ^a^	16.65 ± 0.04 ^b^
ΣSFA	31.73 ± 0.58	31.45 ± 0.06
ΣMUFA	22.81 ± 0.46	23.19 ± 0.10
ΣLC-PUFA	19.30 ± 0.59 ^a^	20.89 ± 0.04 ^b^
20:2n-6/18:2n-6	0.29 ± 0.00 ^a^	0.35 ± 0.01 ^b^
20:3n-3/18:3n-3	0.67 ± 0.00 ^a^	0.69 ± 0.00 ^b^

Results are showed as means ± SEM (*n* = 3). Values in each row with different superscripts indicate significant difference (analyzed by ANOVA followed by paired *t*-test; *p* < 0.05).

**Table 3:** Main fatty acids composition in liver of juvenile rabbitfish injected with control solvent or Hnf4α agonists (Alverine and Benfluorex) (mg·kg^−1^ dry mass).

Main Fatty Acids	0.9% NaCl	DMSO	Alverine	Benfluorex
14:0	64.57 ± 3.00	68.23 ± 6.92	63.33 ± 1.75	66.74 ± 2.42
16:0	665.83 ± 39.65	644.23 ± 66.56	644.91 ± 85.87	632.51 ± 23.44
18:0	135.08 ± 19.18	128.54 ± 5.02	129.51 ± 9.24	149.43 ± 31.85
20:0	11.36 ± 0.74	12.38 ± 0.63	13.16 ± 0.87	12.71 ± 0.46
24:0	5.47 ± 0.43	6.26 ± 1.44	6.99 ± 0.22	6.88 ± 1.66
16:1n-7	127.47 ± 8.56	144.49 ± 17.31	139.50 ± 5.31	137.02 ± 5.78
18:1n-9	498.35 ± 40.16	500.34 ± 18.62	540.13 ± 46.47	508.29 ± 23.00
20:1n-9	6.40 ± 0.81	8.07 ± 0.60	8.45 ± 1.71	8.22 ± 1.75
24:1	5.06 ± 0.64	5.49 ± 0.34	5.77 ± 0.08	5.04 ± 0.47
18:2n-6	166.67 ± 32.11	164.96 ± 17.76	189.71 ± 9.42	215.96 ± 6.42
18:3n-6	10.79 ± 1.13	11.28 ± 1.55	12.72 ± 1.47	14.36 ± 1.08
20:2n-6	10.95 ± 2.11 ^a^	11.91 ± 1.29 ^ab^	14.15 ± 1.29 ^ab^	18.61 ± 1.44 ^b^
20:4n-6 (ARA)	5.81 ± 1.14	6.92 ± 0.39	9.17 ± 0.49	9.09 ± 1.10
22:2n-6	5.10 ± 0.74	5.66 ± 0.27	6.22 ± 0.43	6.72 ± 0.78
18:3n-3	56.06 ± 1.57	58.94 ± 3.93	66.39 ± 2.13	53.71 ± 3.11
18:4n-3	8.92 ± 1.44	12.38 ± 0.63	13.71 ± 0.82	11.44 ± 2.14
20:3n-3	20.89 ± 2.08	27.69 ± 2.82	27.07 ± 1.39	24.82 ± 4.33
20:5n-3 (EPA)	12.89 ± 2.80 ^a^	15.72 ± 2.57 ^ab^	20.24 ± 1.03 ^ab^	21.74 ± 0.80 ^b^
22:5n-3	58.47 ± 14.38	64.67 ± 4.52	87.41 ± 7.27	84.19 ± 11.84
22:6n-3 (DHA)	128.85 ± 32.08 ^a^	150.16 ± 17.21 ^ab^	228.95 ± 21.00 ^b^	184.21 ± 12.77 ^ab^
∑SFA	695.5 ± 50.75	741.14 ± 31.12	703.99 ± 25.38	714.46 ± 54.49
∑MUFA	638.70 ± 49.09	642.50 ± 35.66	688.79 ± 47.88	661.95 ± 17.77
∑LC-PUFA	236.99 ± 54.82 ^a^	282.73 ± 20.30 ^a^	397.02 ± 32.44 ^b^	349.38 ± 22.93 ^ab^

Results are means ± SEM (*n* = 3). Values in each row with different superscripts indicate significant difference (analyzed by ANOVA followed by paired *t*-test; *p* < 0.05).

**Table 4:** PCR primers sequence and RNAi nucleotide sequence used in this study.

Subject	Primers	Nucleotide Sequence
PCR for 5′ flanking sequence cloning	AP1	5′-GTAATACGACTCACTATAGGGC-3′
	AP2	5′-ACTATAGGGCACGCGTGGT-3′
	E5UA0	5′-CCAAACACGTCAAAGGCTAGAGAG-3′
	E5UA1	5′-GTGAAGTACAAAGTGTGGGTGCAG-3′
pfu-PCR for deletion mutant construction	E5P0	5′-CGGGGTACCACCCGCAGTACAAGCAGGAC-3′
	E5P1	5′-CGGGGTACCGTCTGCTTTTAATCGTGTGTTCTGT-3′
	E5P2	5′-CGGGGTACCATCCACAAGATGGCGGTATT-3′
	E5P3	5′-CGGGGTACCGTGCACCTGAGGCTGTACAACT-3′
	E5P4	5′-CGGGGTACCCTGTGATGCTACTCAAAGTTGCTGT-3′
	SigE5UA1	5′-CCGCTCGAGGTGAAGTACAAAGTGTGGGTGCA-3′
EMSA for gel shift	BF (5′ biotinlabeled)	5′-TCTGCACCCACACTTTGTACTTCACCTCG-3′
	BR (5′ biotinlabeled)	5′-CGAGGTGAAGTACAAAGTGTGGGTGCAGA-3′
	UF (5′ unlabeled)	5′-TCTGCACCCACACTTTGTACTTCACCTCG-3′
	UR (5′ unlabeled)	5′-CGAGGTGAAGTACAAAGTGTGGGTGCAGA-3′
RNAi	NC-F	5′-UUCUCCGAACGUGUCACGUTT-3′
	NC-R	5′-ACGUGACACGUUCGGAGAATT-3′
	siRNA-F	5′-AGACUGUAAUUAGACGACAUCTT-3′
	siRNA-R	5′-GAUGUCGUCUAAUUACAGUCUTT-3′
Site-directed mutant construction	Elovl5-D3-M1-F	5′-CGGCATCTCTGCACCCTCACCTCGAGGATATC-3′
	Elovl5-D3-M1-R	5′-GATATCCTCGAGGTGAGGGTGCAGAGATGCCG-3′
	Elovl5-D3-M2-F	5′-TGCCACTCTCCTGCAGGGTCTGCGTGTTCCTC-3′
	Elovl5-D3-M2-R	5′-GAGGAACACGCAGACCCTGCAGGAGAGTGGCA-3′
Hnf4α mRNA construction	T7 promoter primer	5′-TAATACGACTCACTATAGGG-3′
	Pa-Hnf4α	5′-GAAGGAAAAGGCTTCGGAGGGTTGTTA-3′
Q-PCR detection for target gene expression	QS-Hnf4α	5′-CCGACTCTACAGAGCATCACCTG-3′
	QA-Hnf4α	5′-TCATTAGCAGAACCTCCGAGAAG-3′
	QS-Elovl5	5′-GCACTCACCGTTGTGTATCT-3′
	QA-Elovl5	5′-GCAGAGCCAAGCTCATAGAA-3′
	QS-Δ4 Fad	5′-GAACACCATTTGTTCCCGAG-3′
	QA-Δ4 Fad	5′-TTCAGTGCCCTGACGACG-3′
	QS-18S rRNA	5′-CGCCGAGAAGACGATCAAAC-3′
	QA-18S rRNA	5′-TGATCCTTCCGCAGGTTCAC-3′

Restriction sites are underlined: *Kpn*I (GGTACC) and *Xho*I (CTCGAG).

## Abbreviations

ALA	α-linolenic acid (18:3n-3)
ARA	arachidonic acid (20:4n-6)
BHT	2,6-butylated hydroxytoluene
DHA	docosahexaenoic acid (22:6n-3)
DMEM/F12	dulbecco’s modified Eagle’s medium (DMEM)–F12 medium
Elovl	elongases of very long-chain fatty acids
EMSA	electrophoresis mobility shift assay
EPA	eicosapentaenoic acid (20:5n-3)
Fad	fatty acyl desaturases
FAME	fatty acid methyl esters
FBS	fetal bovine serum
FM	fish meal
FO	fish oil
GC-MS	Gas Chromatography-Mass Spectrometer
HEK293T cell	human embryonic kidney cell line
Hnf4α	hepatocyte nuclear factor 4α
NF-Y	nuclear factor Y
LA	linoleic acid
LC-MS	liquid chromatography coupled with tandem mass spectrometry
LC-PUFA	long-chain polyunsaturated fatty acids
LXR	liver X receptor
SCHL	*Siganus canaliculatus* hepatocytes cell line
siRNA	small interfering RNA
SRE	sterol regulatory element
SREBPs	sterol regulatory element binding proteins
TSS	transcription start site
VO	vegetable oil
